# Outcome in acute ischemic stroke patients with large-vessel occlusion and initial mild deficits

**DOI:** 10.3389/fstro.2024.1426084

**Published:** 2024-07-02

**Authors:** Jacob S. Kazmi, Joseph O'Hara, Amir Gandomi, Jason J. Wang, Maria X. Sanmartin, Bo Yang, Pina C. Sanelli, Jeffrey M. Katz

**Affiliations:** ^1^Northwell, New Hyde Park, NY, United States; ^2^Donald and Barbara Zucker School of Medicine at Hofstra/Northwell, Hempstead, NY, United States; ^3^Frank G Zarb School of Business, Hofstra University, Hempstead, NY, United States; ^4^Institute of Health System Science, The Feinstein Institutes for Medical Research, Manhasset, NY, United States; ^5^Department of Radiology, Donald and Barbara Zucker School of Medicine at Hofstra/Northwell, Hempstead, NY, United States; ^6^Department of Neurology, Donald and Barbara Zucker School of Medicine at Hofstra/Northwell, Hempstead, NY, United States

**Keywords:** ischemic stroke, low NIHSS, early neurological deterioration, predictors, functional status

## Abstract

**Background:**

The management of patients with initially mild acute ischemic stroke (AIS), defined by the National Institutes of Health Stroke Scale (NIHSS) scores 0–5, remains ambiguous despite advances in stroke treatment. The early identification of patients likely to deteriorate is critical in preventing lasting disability.

**Aims:**

We investigated the frequency and early predictors of poor functional outcomes in AIS patients with large-vessel occlusion (LVO) and initial mild deficits.

**Methods:**

We performed a retrospective observational study of consecutive AIS patients admitted to a single comprehensive stroke center between 2018 and 2021. The inclusion criteria were a diagnosis of AIS, an arrival NIHSS score of 0–5, imaging-confirmed LVO, and arrival within 24 h of the last-known-well time. The primary outcome was the change in the discharge-modified Rankin Score (ΔmRS) from baseline, categorized as 0–1 (stable outcome) or >1 (poor outcome). Early neurological deterioration was defined as a mean NIHSS score increase of >1 in the first 24-h period. Univariate and multivariable regression analyses were performed. The mean daily NIHSS scores were compared between groups using an analysis of variance (ANOVA).

**Results:**

Of 4,410 stroke admissions, 120 patients met the study inclusion criteria, with 71 (59.2%) patients having a ΔmRS of 0–1 and 49 (40.8%) patients having a ΔmRS of > 1. The mean arrival NIHSS score was similar between groups. However, the mean first-24-h NIHSS score was significantly higher in the poor outcome group vs. the stable outcome group (2.13 vs. 0.95, *p* < 0.001). A demographic-adjusted multivariable logistic regression revealed that a higher mean first-24-h NIHSS score was the sole early predictor of poor outcome (odds ratio [OR] of 1.65 and a 95% confidence interval [CI] of [1.18, 2.48]). The only association with early neurological deterioration was vertebral artery occlusion, with an OR of 0.35 and a 95% CI of [0.14, 0.81]. The trending mean daily NIHSS scores revealed that patients with poor outcomes deteriorate within 24 h, a significant difference from the stable group (*p* < 0.001).

**Conclusion:**

Poor outcomes occurred in a significant proportion of LVO patients with initial mild deficits. The only association was early neurological deterioration. To prevent poor outcomes, rapid identification of any clinical deterioration should prompt consideration of thrombectomy.

## 1 Introduction

Treatment of acute ischemic stroke (AIS) has been revolutionized over the past decade through developments in thrombolytic therapies, the evolution and expansion of endovascular thrombectomy (EVT) techniques, neuroimaging advancements, and enhancements in acute stroke workflow efficiencies (Goyal et al., 2016; Tsivgoulis et al., 2023). However, managing patients with mild strokes, defined by the National Institutes of Health Stroke Scale (NIHSS) score of < 6, remains ambiguous, especially for patients with documented large-vessel occlusion (LVO). Currently, clinicians approach treatment heterogeneously and on a case-by-case basis (McDonough et al., 2021; Brake et al., 2024). Recent meta-analyses have not demonstrated clear superiority among treatment options for mild AIS-LVO patients, such as EVT, intravenous thrombolysis, and conservative medical management (Volny et al., 2020; McCarthy et al., 2021; Abecassis et al., 2023; Safouris et al., 2023).

Since its introduction over 25 years ago, the NIHSS has been used as a triage measure, prognostic indicator, and treatment selection tool (Adams et al., 1993; Schlegel et al., 2003; Klingman et al., 2021; Tramonte et al., 2022). However, approximately one-third of AIS patients classified as mild by the NIHSS on hospital arrival experience lasting disability, underscoring the need for rapid identification of patients at risk of early neurological deterioration (END; Khatri et al., 2012; Asdaghi et al., 2019). END is traditionally defined as a ΔNIHSS of ≥ 4 points within 24 h. However, previous studies of patients with mild AIS (arrival NIHSS score of 0–5) have utilized a ΔNIHSS of ≥ 1 or 2 points (Heldner et al., 2015; Haussen et al., 2017). Estimates of END incidence in mild AIS patients are between 20% and 40%, which is thought to result from progressive ischemia rather than hemorrhagic transformation (Seners and Baron, 2018; Volbers et al., 2021). A strong association between END and lasting disability in mild AIS-LVO patients was demonstrated from the Safe Implementation of Thrombolysis in Stroke (SITS) International Stroke Thrombolysis Register data, which revealed that 77% were either deceased or functionally dependent at 3-month follow-up (Mazya et al., 2018).

Yet, poor functional outcomes in AIS-LVO patients with initial low NIHSS scores are challenging to predict. Herein, we investigated mean daily NIHSS scores during hospitalization in mild AIS-LVO patients, defined as arrival NIHSS score of 0–5 with computed tomography angiography–confirmed LVO. Our goal was to determine early predictive factors of poor functional outcomes, focusing on the role of END within the first 24 h—the current time window in which EVT may be performed to improve outcomes. Our main outcome measure was the change from preadmission baseline modified Rankin Scale (mRS) to hospital-discharge mRS (ΔmRS), defining a stable outcome as ΔmRS 0–1 and a poor outcome as ΔmRS > 1. Our primary objectives were to define the frequency and early predictors of poor outcomes in mild AIS-LVO patients. We hypothesized that early neurological deterioration is the strongest predictor of a poor outcome in mild AIS-LVO patients.

## 2 Materials and methods

### 2.1 Study population

We utilized the Stroke Health Outcomes (SHOUT) database—a retrospective data set of consecutive AIS patients from a single comprehensive stroke center in the New York City area. The database contains all patients aged 18 years or older who were discharged with a stroke diagnosis. The data in the SHOUT database are imported from the Get With The Guidelines—Stroke database and are supplemented with imaging data extracted from electronic health records. The inclusion criteria for this study were all patients who had (1) a discharge diagnosis of AIS between December 2018 and December 2021, (2) an arrival NIHSS score of between 0 and 5 upon presentation to the emergency department, (3) imaging-confirmed LVO based on computed tomography angiography (defined as internal carotid artery terminus, middle cerebral artery M1 or M1-like [both M2] segments, basilar artery, and vertebral artery); and (4) presented with a last-known-well time to hospital arrival time (LKWA) of < 24 h. The exclusion criteria were a discharge diagnosis of hemorrhagic stroke or transient ischemic attack, no NIHSS score recorded on hospital arrival, or no recorded discharge mRS score. All patients were evaluated, diagnosed, and treated per institutional protocols at the time of hospitalization. This study was approved by the institutional review board of the Feinstein Institutes for Medical Research [22-0955], with a waiver of consent given the retrospective nature of the study.

### 2.2 Data collection

The demographic variables included age, gender, and self-reported race (white, Black, Asian, mixed/other). The clinical variables included medical comorbidities and stroke risk factors (atrial fibrillation, dyslipidemia, coronary artery disease or previous myocardial infarction, diabetes mellitus, smoking status, obesity, hypertension, previous stroke, and family history of stroke), baseline (pre-admission) mRS score, LKWA time, and arrival NIHSS score. Ambulatory status before admission, defined as the ability to walk independently without a device or assistance, and independent ambulation on admission, defined as the ability to walk without assistance from another person upon arrival to the hospital, were also recorded. The treatment variables included intravenous thrombolysis (with alteplase during the study period), EVT, and no acute treatment. Stroke etiology was defined according to Trial of Org 10172 in Acute Stroke Treatment (TOAST) criteria and dichotomized as atherosclerosis/large artery dissection or embolism/cryptogenic to reflect pathophysiologic mechanism (Adams et al., 1999).

All individual patient NIHSS scores were collected for up to 10 days of hospitalization. The frequency of NIHSS assessments varied depending on the hospital unit (i.e., emergency department, neurosciences intensive care unit, stroke unit, or regular floor), day of hospitalization, and clinical status. To compare changes in stroke severity by day, the mean daily NIHSS score was calculated for every 24-h period after arrival for each patient from the recorded values in electronic medical records. END was defined as an increase in the mean first-24-h NIHSS score from the arrival NIHSS score of >1 point.

### 2.3 Imaging data

The imaging data included the stenosis/occlusion locations collected from the neuroradiology reports before any treatment. Tandem occlusion was defined as the coexistence of extracranial internal carotid artery occlusion with either intracranial internal carotid artery, M1 middle cerebral artery, or M1-like (both M2 trunks) occlusions (Jadhav et al., 2019). Computed tomography perfusion (CTP) data were extracted from RAPID AI Software (iSchemaView, Menlo Park, CA) results. When multiple CTP studies were performed for a single patient, the last imaging study performed in the first 24 h after arrival and before treatment was used. In the event of a two-slab acquisition, the parameters of each slab were averaged. Core infarct volume on CTP imaging was defined as tissue volume with relative cerebral blood flow < 30%. The hypoperfusion intensity ratio was calculated as T_max_ > 6 s/T_max_ > 10 s.

### 2.4 Discharge outcome

The outcome variables included discharge mRS score, previously shown to be predictive of 90-day mRS scores (ElHabr et al., 2021), and in-hospital mortality. To determine the impact of the stroke event on each individual's functional neurological outcome, our primary outcome measure was the ΔmRS score, calculated for each patient by subtracting the discharge mRS score from the baseline mRS score. The baseline mRS scores were taken from the attending neurologist's notes on admission when available. For cases where no baseline mRS score was documented, an estimate was made based on the previous level of function data contained in physical and occupational therapy records, using standard scoring criteria (van Swieten et al., 1988; Sulter et al., 1999). Patients were grouped for analysis as either ΔmRS 0–1 point (stable functional outcome) or ΔmRS > 1 point (poor functional outcome). Patients calculated to have improvement in ΔmRS were grouped with the ΔmRS 0–1 group.

### 2.5 Statistical analyses

Descriptive statistics for patients' demographic, clinical, imaging, and treatment data were summarized using mean with standard deviation, median with a minimum–maximum range, or frequency count when appropriate. The parametric data were compared between stable (ΔmRS 0–1) and poor functional (ΔmRS >1) outcome groups using independent, two-tailed Student's *t*-tests, and non-parametric data were compared using the Mann–Whitney *U* test. The categorical variables were compared between groups using Pearson's chi-squared test with Yate's continuity correction applied when appropriate. The mean daily NIHSS data were compared using two-way repeated measures analysis of variance (ANOVA), and 95% confidence intervals (CIs) were calculated from standard error.

To assess the association between the mean first-24-h NIHSS score and poor functional outcome (ΔmRS > 1), logistic regression analyses were utilized in a stepwise manner. Univariate binary logistic regression was performed with a ΔmRS of > 1 as the dependent variable and demographic, clinical, and imaging characteristics as the independent variables. Independent variables were not included if there were fewer than two patients in either outcome group. Each independent variable that passed the significance threshold of *p* < 0.10 was entered into a multivariable logistic regression model, where ORs, 95% CIs, and *p*-values were calculated. To assess the association between specific demographic, clinical, and imaging factors with END, a similar stepwise approach was employed in a separate multivariable logistic regression model with END as the dependent variable. The NIHSS score was treated as a continuous variable in all analyses.

Given the sample size of 120 patients and few remaining independent variables after stepwise exclusion, the multivariable regression models were adequately powered. For multiple regressions, *p* < 0.05 were considered statistically significant. All statistical analyses were performed using the R statistical computing language (v 4.3.2).

## 3 Results

### 3.1 Study cohort characteristics

**Figure 1:**
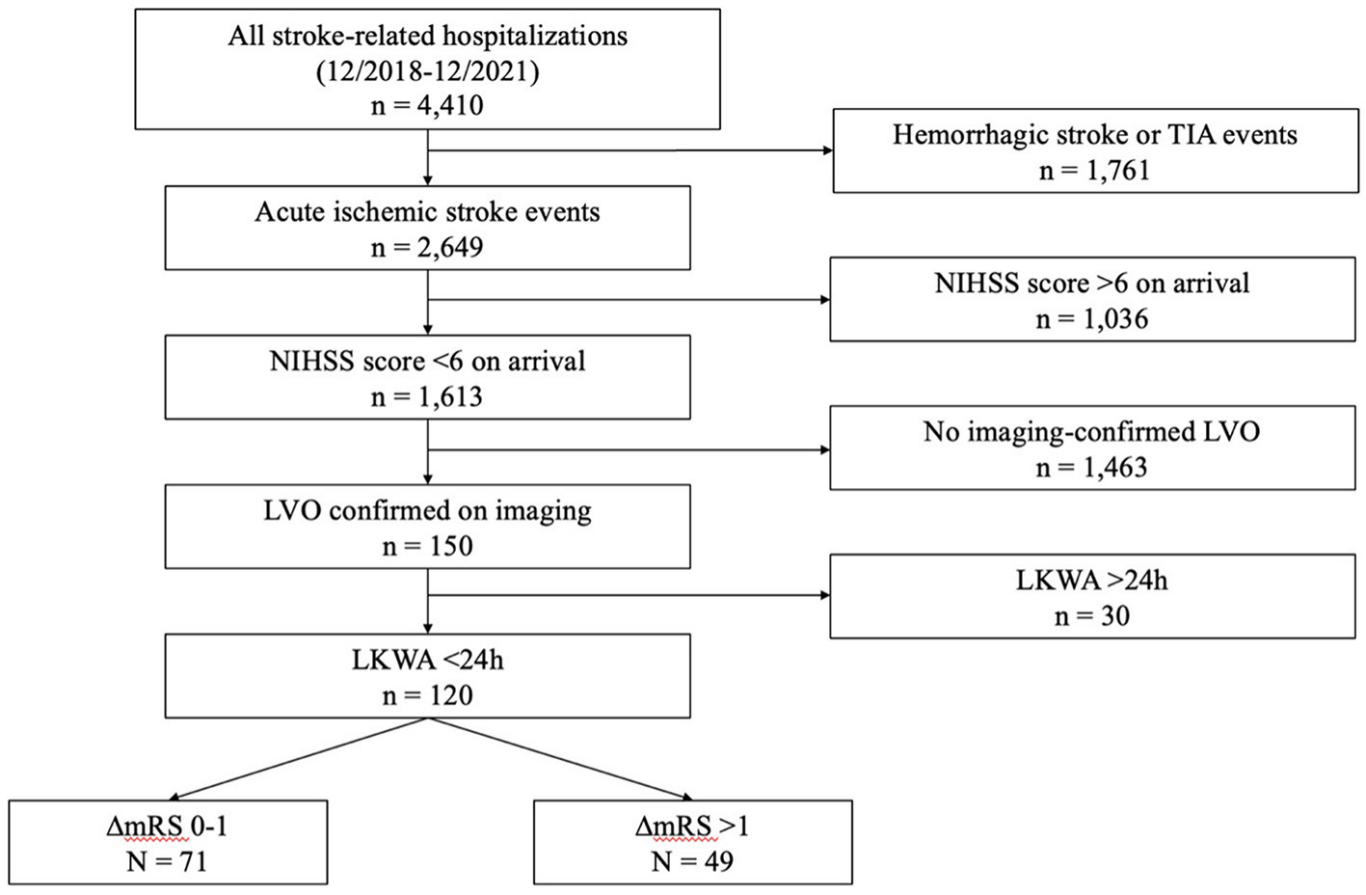
Study flowchart. TIA, transient ischemic attack; NIHSS, National Institutes of Health Stroke Scale; LKWA, last-known-well-to-arrival time; mRS, modified Rankin Scale.

Of 4,410 stroke episodes in the SHOUT database between December 2018 and December 2021, 120 patients met study inclusion (Figure 1). The mean (± standard deviation) age was 65.8 (±14.6) years, 72 (60.0%) patients were male, the mean arrival NIHSS score was 2.22 (±1.67), and the mean LKWA time of 7:38 (±6:52) h (Table 1). A total of 58 (49%) patients had CTP studies performed that were used in subsequent analyses. Intravenous thrombolysis was administered to 16 (13.3%) patients, all upon hospital arrival, while EVT was performed in 9 (7.5%) patients, 6 of whom at initial presentation. The mean mRS at discharge was 2.03 (±1.48), and in-hospital mortality occurred in 4 (3.3%) patients.

**Table 1:** Baseline patient characteristics, treatment utilization, and mortality data between groups.

	**Total (*n* = 120)**	**ΔmRS 0–1 (*n* = 71)**	**ΔmRS > 1 (*n* = 49)**	***p*-value**
Male (*n*, %)	72 (60.0)	42 (59.2)	30 (61.2)	0.969
Age (year, *SD*)	65.8 ± 14.6	66.2 ± 14.1	65.2 ± 15.5	0.728
**Race**				0.663
Black (*n*, %)	20 (16.7)	11 (15.5)	9 (18.4)	
White (*n*, %)	63 (52.5)	41 (57.7)	22 (44.9)	
Asian (*n*, %)	13 (10.8)	8 (11.3)	6 (10.2)	
Mixed/Other (*n*, %)	12 (10.0)	4 (5.6)	8 (16.3)	
Unreported (*n*, %)	14 (11.7)	8 (11.3)	6 (12.2)	
Arrival NIHSS Score (mean, *SD*)	2.22 ± 1.67	2.11 ± 1.75	2.37 ± 1.55	0.384
Mean First 24-h NIHSS (mean, *SD*)	1.43 ± 1.95	0.95 ± 1.11	2.13 ± 2.62	< 0.001
LKWA (h, *SD*)	7.6 ± 6.9	7.8 ± 7.0	7.4 ± 6.8	0.853
Ambulatory Prior to Stroke (*n*, %)	94 (78.3)	56 (78.9)	38 (77.6)	0.400
Independent Ambulation on Admission (*n*, %)	42 (35.0)	29 (40.8)	13 (26.5)	0.155
Atrial Fibrillation (*n*, %)	14 (11.7)	10 (14.1)	4 (8.2)	0.482
Smoking (*n*, %)	13 (10.8)	6 (8.5)	7 (14.3)	0.476
Coronary Artery Disease/Past MI (*n*, %)	15 (12.5)	7 (9.9)	8 (16.3)	0.440
Dyslipidemia (*n*, %)	44 (36.7)	27 (38.0)	17 (34.7)	0.857
Obesity (*n*, %)	59 (49.2)	40 (56.3)	19 (38.8)	0.088
Diabetes Mellitus (*n*, %)	31 (25.8)	17 (23.9)	14 (28.6)	0.721
Previous Stroke (*n*, %)	15 (12.5)	7 (9.9)	8 (16.3)	0.440
Family History of Stroke (*n*, %)	1 (0.8)	1 (1.4)	0	0.999
Hypertension (*n*, %)	85 (70.8)	49 (69.0)	36 (73.5)	0.746
Acute Infarct on Imaging Studies (*n*, %)	105 (87.5)	62 (87.3)	43 (87.8)	0.932
**Mechanism**				0.975
Atherosclerotic/Dissection (*n*, %)	50 (41.7)	29 (41.7)	21 (42.9)	
Cryptogenic (*n*, %)	70 (58.3)	42 (59.2)	28 (57.1)	
**LVO location**				0.381
ICA (*n*, %)	12 (10.0)	9 (12.6)	3 (6.1)	
MCA (*n*, %)	54 (45.0)	35 (49.3)	19 (38.8)	
Basilar Artery (*n*, %)	11 (9.2)	5 (7.0)	6 (12.2)	
Vertebral Artery (*n*, %)	36 (30.0)	19 (26.8)	17 (34.7)	
Tandem Occlusion (*n*, %)	7 (5.8)	3 (4.2)	4 (8.2)	
Multiple Occlusions (*n*, %)	12 (10.0)	5 (7.0)	7 (14.3)	0.624
Intravenous Thrombolysis (*n*, %)	16 (13.3)	8 (11.3)	8 (16.3)	0.846
Mechanical Thrombectomy (*n*, %)	9 (7.5)	5 (7.0)	4 (8.2)	0.999

NIHSS, National Institutes of Health Stroke Scale; LKWA, last-known-well-to-arrival time; MI, myocardial infarction; ICA, internal carotid artery; MCA, middle cerebral artery.

Among the 120 patients, poor outcomes (ΔmRS > 1) occurred in 49 (40.8%) patients, and 71 (59.2) patients had stable outcomes (ΔmRS 0–1). Clinical, imaging, and treatment factors are compared between groups in Table 1, and CTP parameter comparisons between groups are shown in the Supplementary Table S1. We found no difference in the mean arrival NIHSS scores between groups. However, the mean first-24-h NIHSS scores were significantly higher in the poor outcome group than the stable outcome group (2.13 vs. 0.95, *p* < 0.001), indicating END. We found no other significant differences between outcome groups in terms of demographics, stroke risk factors, imaging findings, or treatment utilization. Endovascular thrombectomy was performed upon neurological worsening in one of four patients in the poor-outcome group and in two of five patients in the stable-outcome group, while the remaining patients received reperfusion therapy upon hospital arrival.

### 3.2 Associations with poor functional outcome

The univariate analysis revealed that the arrival NIHSS score had no association with functional outcome (*p* = 0.314; see Supplementary Table S2). However, a higher mean first-24-h NIHSS score, indicative of END, was found to significantly increase the odds of poor outcome (OR 1.47, 95% CI [1.15, 2.00]). The multivariable logistic regression model, using variables found to impact functional outcome in the univariate analysis and adjusted to account for patient age, gender, race, and occluded vessel location (Table 2), revealed that a higher mean first-24-h NIHSS score increased the odds of poor outcome (adjusted OR 1.65, 95% CI [1.18, 2.48]). No other associations with poor outcome were found.

**Table 2:** Multivariable logistic regression model (dependent variable: ΔmRS of > 1) adjusted for age, gender, race, and occlusion location.

	**OR**	**95% CI**		***p*-value**
Obesity	0.50	0.19	1.29	0.155
Unable to ambulate on admission	1.58	0.90	2.82	0.114
Mean First 24-h NIHSS scores	1.65	1.18	2.48	0.009

Odds ratio (OR) and 95% confidence interval (CI) are presented as odds of achieving a ΔmRS of > 1 point, or poor functional outcome, at the time of discharge. NIHSS, National Institutes of Health Stroke Scale.

### 3.3 Associations with END

For this analysis, END was defined as the increase in the mean first-24-h NIHSS score from the arrival NIHSS score of >1 point. Of the 116 patients with available average daily NIHSS scores, 53 (45.7%) were categorized as having END, and 63 (54.3%) patients had no END. The univariate logistic regression analysis revealed that only higher arrival NIHSS score (OR 2.03, 95% CI [1.55, 2.75]) and vertebral artery occlusion (OR 0.35, 95% CI [0.14, 0.81]) were significantly associated with END (Supplementary Table S3). The arrival NIHSS scores were not included in multivariable models given their close association with mean first-24-h NIHSS scores. A demographic-adjusted multivariable regression analysis revealed that vertebral artery occlusion significantly decreased the odds of END [OR 0.31, 95% CI (0.12, 0.78); Table 3].

**Table 3:** Multivariable logistic regression model (dependent variable: mean first-24-h NIHSS score of > 1) adjusted for age, gender, and race.

	**OR**	**95% CI**		***p*-value**
Vertebral artery occlusion	0.31	0.12	0.78	0.015

Odds ratio (OR) and 95% confidence interval (CI) are presented as odds of achieving a mean first-24-h National Institutes of Health Stroke Scale (NIHSS) of > 1.

### 3.4 NIHSS scores over the hospital course

**Figure 2:**
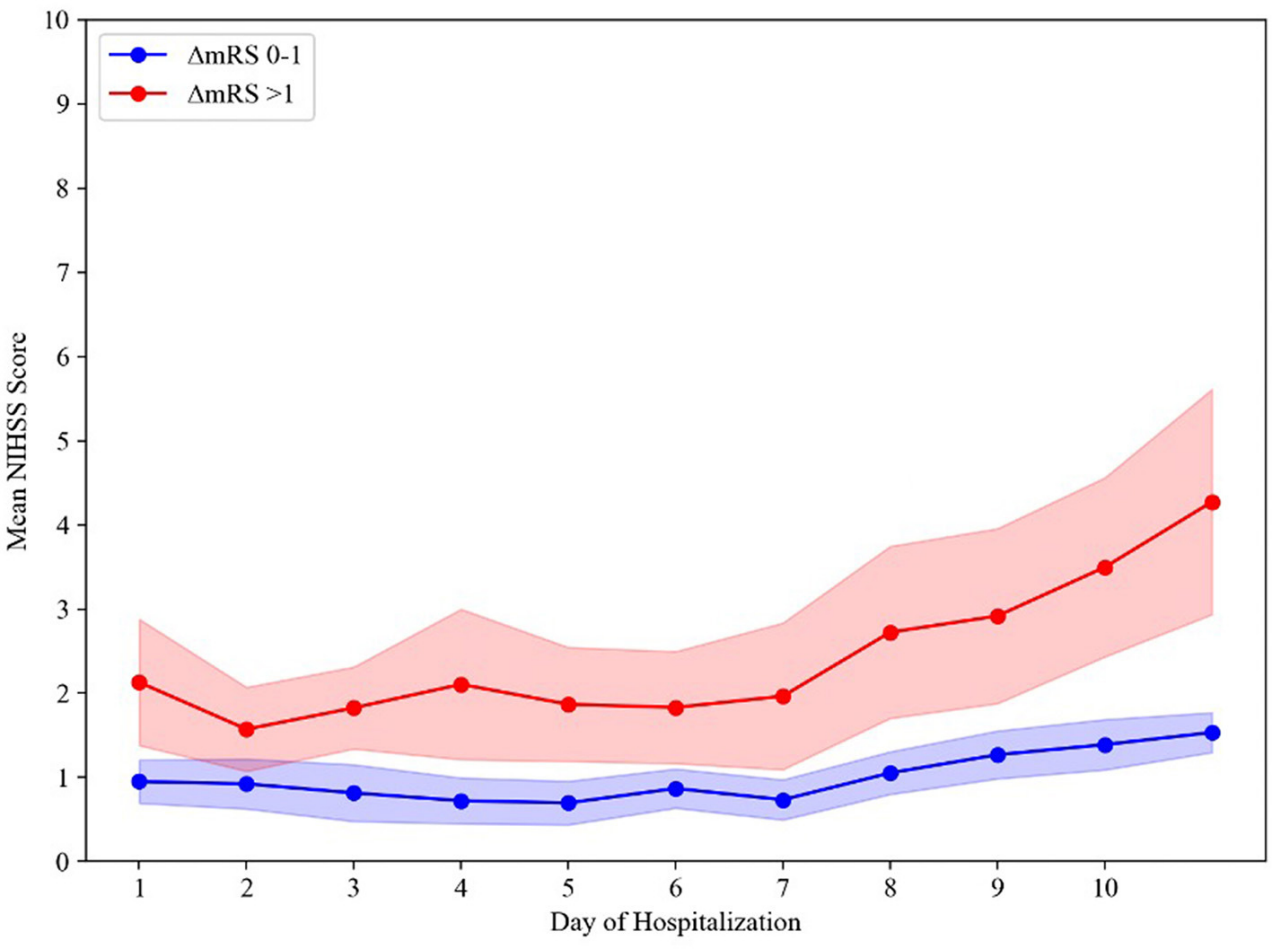
Time course of mean daily NIHSS scores between groups. The shading represents a 95% confidence interval. ΔmRS, change in modified Rankin Scale score from preadmission baseline function to hospital discharge.

Comparing daily mean NIHSS scores (available for 116/120 [96.6%] patients) during hospitalization (Figure 2) found that on average, stable- (ΔmRS 0–1) and poor-outcome (ΔmRS > 1) groups' daily mean NIHSS scores diverged in the first 24 h of admission, again indicating END. For patients in the stable outcome group, the daily mean NIHSS scores remained relatively constant, with NIHSS scores below 2 throughout the hospitalization. Mean daily NIHSS scores were consistently higher for the poor outcome group compared to the stable group. Major uptrends for the poor outcome group occurred within 24 h (END) and again at 7 days. A two-way ANOVA showed a main effect of hospitalization day (*p* < 0.001) and a main effect of the functional outcome group (*p* < 0.001) on mean daily NIHSS scores. We additionally observed a significant interaction between the day of hospitalization and functional outcome (*p* < 0.001), indicating that mean daily NIHSS scores changed in a significantly different manner between groups over the course of hospitalization.

## 4 Discussion

In this retrospective study of initial mild AIS-LVO patients, we found that 40.8% of patients were discharged with a poor functional outcome, defined as an increase in discharge mRS from baseline of >1 point, and 3.3% had in-hospital mortality. This is consistent with previous studies of mild AIS-LVO patients and underscores the need for early identification of those at risk for developing lasting disability (Khatri et al., 2012; Klingman et al., 2021; Volbers et al., 2021). Currently, no standardized guidance for the treatment of mild AIS-LVO patients exists, and meta-analyses have not determined that intravenous thrombolysis or EVT is indicated for treating these patients. Currently, clinical trials of thrombectomy in LVO patients with low NIHSS scores, including the Endovascular Therapy for Low NIHSS Ischemic Strokes (ENDOLOW, NCT04167527) and Minor Stroke Therapy Evaluation (MOSTE, NCT03796468) randomized clinical trials, are ongoing.

Our results indicate that END within the first 24 h of hospital arrival is associated with poor discharge outcomes and supports the existing notion that stroke patients who deteriorate early in their stroke course are more likely to have lasting disability (Jadhav et al., 2019; Klingman et al., 2021; Volbers et al., 2021). Furthermore, our findings demonstrate that even minor increases in the NIHSS scores in mild AIS-LVO patients may be clinically relevant and should prompt consideration of EVT even if the NIHSS score remains low (0–5 range). In our study, only isolated vertebral artery occlusion was associated with END (decreased odds), perhaps because patients with posterior inferior cerebellar infarctions who deteriorate do so beyond 24 h secondary to cerebral edema, not to the perfusion failure that underlies END in other LVO locations. A decreased odds of END associated with vertebral artery occlusion was previously reported by others in a similar study that included 88 AIS-LVO patients with an arrival NIHSS score of < 5 (Volbers et al., 2021). These authors additionally found that higher baseline mRS and arrival NIHSS scores and an LVO location in the M1 segment increased the risk of END. In a retrospective, multicenter analysis of 729 French mild AIS-LVO patients, occlusions in the internal carotid artery were associated with END (Seners et al., 2021). Additionally, a retrospective analysis of 1,086 mild AIS-LVO patients from Korea found that higher arrival NIHSS scores, extracranial internal carotid artery involvement, tandem occlusion, and preexisting hypertension significantly increased the odds of END (Kim et al., 2013; Jadhav et al., 2019). Our study found no other significant factors associated with either END or a poor outcome, including CTP parameters that have not been previously reported in this population. Given our finding that only END was associated with poor outcomes and the absence of reproducible predictors of END, our results reinforce the importance of frequent serial NIHSS assessments during the first 24 h of hospital admission for all mild AIS-LVO patients, and the potential importance of expeditious EVT for those who have even mild clinical deterioration. It is important to note, however, that the benefits of thrombectomy in this population are speculative since rescue thrombectomy has not been proven beneficial in mild AIS-LVO patients, and at least some data suggest it may be harmful (Volbers et al., 2021; Kim et al., 2022).

Trending daily mean NIHSS scores show that between 1 and 7 days, the mean daily NIHSS scores remain relatively constant for both the stable- and poor-outcome groups. However, the daily mean NIHSS scores increased in both groups beyond 7 days and more acutely in the poor-outcome group. This observation may be related to the presence of hospital-acquired medical complications in the poor-outcome group and the influence that these complications have on the NIHSS. Systemic infections, pneumonia, and other metabolic disturbances are known to worsen the neurological examination due to metabolic encephalopathy (Ghelani et al., 2021; Pohl et al., 2021). These, and other complications, such as dysphagia, diarrhea/constipation, delirium, and venous thromboembolism, further prolong AIS patient hospitalization duration (Gaspari et al., 2019; Wang et al., 2023). As a result, patients may experience a delay in the initiation of physical rehabilitation, which is associated with worse functional outcomes (Wang et al., 2022). Thus, our results suggest the importance of both mitigating hospital-acquired medical complications and expeditious discharge of mild AIS-LVO patients to home or an acute rehabilitation facility before the development of medical complications and deconditioning (Kumar et al., 2020).

Our study has several strengths and limitations. The breadth of imaging parameters and clinical factors included in our analyses is unique relative to similar studies with larger sample sizes and is a strength of our study. The quantity and granularity of variables evaluated enabled a robust analysis of many relevant confounders that have not previously been investigated as predictors of poor outcomes in patients with LVO initially presenting with an NIHSS score of < 6. To our knowledge, no previous studies have examined mean daily NIHSS scores among patients in this population. Yet, our study is retrospective, and as a result, the study cohort is heterogeneous with respect to imaging studies performed, imaging acquisition parameters obtained, and treatments provided. Moreover, we could not utilize the 90-day mRS as an outcome measure because this is not consistently collected in the SHOUT database. Given the sample size of our study, future studies with larger cohorts may be able to capture additional early predictors.

A significant proportion of mild AIS-LVO patients had poor functional outcomes at hospital discharge. A rising NIHSS score in the first 24 h of hospitalization, indicative of END, was the only early factor associated with poor functional outcome. This supports the close monitoring of mild AIS-LVO patients and the consideration of reperfusion therapy should repeat NIHSS scores after hospital arrival increase, even if the patient remains in the “mild” stroke range of NIHSS scores 0–5, although the benefits of this approach have yet to be proven in prospective randomized clinical trials. A trending daily mean NIHSS score over the hospital course also suggests that prolonged hospitalization, which may be related to hospital-acquired medical complications, may contribute to poor outcomes at discharge in this population. Future studies should better elucidate these hospital course factors on the outcome and attempt to distinguish whether specific deficits on the arrival NIHSS are more predictive of poor outcomes in the mild NIHSS range. Furthermore, prospective studies of advanced imaging parameters specific to the robustness of collateral flow on multiphase computed tomography angiography and CTP are warranted.

## Data availability statement

The raw data supporting the conclusions of this article will be made available by the authors, without undue reservation.

## Ethics statement

The studies involving humans were approved by Institutional Review Board of the Feinstein Institutes for Medical Research [22-0955]. The studies were conducted in accordance with the local legislation and institutional requirements. Written informed consent for participation was not required from the participants or the participants' legal guardians/next of kin in accordance with the national legislation and institutional requirements.

## Author contributions

JKaz: Conceptualization, Data curation, Formal analysis, Investigation, Writing – original draft. JO'H: Data curation, Resources, Writing – review & editing. AG: Data curation, Formal analysis, Visualization, Writing – review & editing. JW: Data curation, Formal analysis, Methodology, Resources, Writing – review & editing. MS: Writing – review & editing. BY: Data curation, Resources, Writing – review & editing. PS: Conceptualization, Investigation, Methodology, Supervision, Writing – review & editing. JKat: Conceptualization, Investigation, Supervision, Writing – original draft, Writing – review & editing.
