# Supplementary material for: Outcome in acute ischemic stroke patients with large-vessel occlusion and initial mild deficits
Source: Front Stroke. 2024 Jul 2;3:1426084. doi: 10.3389/fstro.2024.1426084 (PMC12802615; doi:10.3389/fstro.2024.1426084)
Supplement: Supplementary file 1 [file Data_Sheet_1.PDF]

Supplemental Table 1. Computed tomography perfusion (CTP) results for patients in the study that received perfusion imaging.

Perfusion Data	Total (n = 58)	ΔmRS 0-1 (n = 37)	ΔmRS>1 (n = 21)	P-value
**CBF < 20% Volume** (mL, SD)	1.05 ± 5.85	0.22 ± 1.32	2.52 ± 9.53	0.282
**CBF < 30% Volume** (mL, SD)	5.07 ± 13.02	3.08 ± 7.17	8.57 ± 19.24	0.220
**CBF < 34% Volume** (mL, SD)	6.97 ± 16.55	4.22 ± 8.96	11.81 ± 24.44	0.183
**CBF < 38% Volume** (mL, SD)	9.26 ± 19.97	6.14 ± 11.34	14.76 ± 29.24	0.207
**TMAX > 4 s Volume** (mL, SD)	180.78 ± 154.80	179.00 ± 154.80	183.90 ± 150.96	0.908
**TMAX > 6 s Volume** (mL, SD)	71.52 ± 74.95	70.16 ± 65.28	73.90 ± 91.27	0.870
**TMAX > 8 s Volume** (mL, SD)	39.45 ± 54.40	35.14 ± 42.79	47.05 ± 71.02	0.490
**TMAX > 10 s Volume** (mL, SD)	24.07 ± 41.79	20.73 ± 29.77	29.95 ± 57.67	0.501
**Mismatch Volume** (mL, SD)	67.07 ± 71.62	67.08 ± 64.37	67.05 ± 84.62	0.999
**Hypoperfusion Intensity Ratio** (mean, SD)	0.24 ± 0.24	0.26 ± 0.22	0.22 ± 0.26	0.619

CBF, cerebral blood flow; ΔmRS, change in modified Rankin Scale score.

Supplemental Table 2. Univariate logistic regression showing odds of ΔmRS > 1 point at time of discharge.

	OR	95% CI		P-Value
**Male Sex**	1.05	0.50	2.21	0.894
**Age > 65 years**	0.94	0.45	1.96	0.871
**Black or African American**	1.44	0.53	3.84	0.461
**White**	0.68	0.32	1.47	0.327
**Asian**	0.91	0.26	2.83	0.869
**Arrival NIHSS**	1.12	0.90	1.40	0.314
**Mean Frst 24-hour NIHSS Scores**	1.47	1.15	2.00	0.007
**Ambulatory Prior to Stroke**	2.51	0.71	11.8	0.181
**Independent Ambulation on Admission**	1.58	1.07	2.41	0.026
**Atrial Fibrillation**	0.56	0.15	1.78	0.346
**Microvascular Disease (Radiologic)**	1.66	0.78	3.79	0.195
**Smoking**	1.82	0.61	5.56	0.278
**Coronary Artery Disease/Past MI**	1.82	0.61	5.56	0.278
**Dyslipidemia**	0.90	0.42	1.90	0.776
**Obesity**	0.47	0.22	0.96	0.042
**Diabetes Mellitus**	1.30	0.57	2.97	0.527
**Previous Stroke**	1.57	0.54	4.58	0.400
**Hypertension**	1.37	0.62	3.09	0.443
**CTP Core Infarct**	1.22	0.39	4.19	0.738
**Atherosclerotic/Dissection**	1.06	0.51	2.20	0.871
**Embolic/Cryptogenic**	0.94	0.45	1.96	0.871
**Extracranial ICA**	0.71	0.21	2.14	0.558
**Intracranial ICA**	0.78	0.25	2.21	0.650
**MCA**	0.83	0.40	1.69	0.608
**Basilar Artery**	1.59	0.57	4.38	0.368
**Vertebral Artery**	1.52	0.70	3.30	0.289
**Tandem Occlusion**	1.23	0.29	4.88	0.770
**Any tPA**	1.81	0.70	4.72	0.220
**Intravenous tPA**	1.57	0.54	4.58	0.400
**Mechanical Thrombectomy**	1.20	0.28	4.77	0.793

OR, odds ratio; CI, Confidence Interval; MI, myocardial infarction; ICA, internal carotid artery; ACA, anterior cerebral artery; MCA, middle cerebral artery; PCA, posterior cerebral artery; tPA, tissue-type plasminogen activator.

Supplemental Table 3. Univariate logistic regression showing odds of ΔmRS > 1 point at time of discharge.

	OR	95% CI		P-Value
**Arrival NIHSS**	2.03	1.55	2.75	< 0.001
**Vertebral Artery Occlusion**	0.35	0.14	0.81	0.017

OR, odds ratio; CI, Confidence Interval; NIHSS, National Institutes of Health Stroke Scale Score.
